# Comorbidity patterns and socioeconomic inequalities in children under 15 with medical complexity: a population-based study

**DOI:** 10.1186/s12887-020-02253-z

**Published:** 2020-07-30

**Authors:** Neus Carrilero, Albert Dalmau-Bueno, Anna García-Altés

**Affiliations:** 1 Agència de Qualitat i Avaluació Sanitàries de Catalunya (AQuAS), Barcelona, Spain; 2 Department of Experimental and Health Sciences (DCEXS), Universitat Pompeu Fabra, Barcelona, Spain; 3 Institut de Recerda de l’Hospital de la Santa Creu i Sant Pau (IR Sant Pau), Barcelona, Spain; 4 CIBER de Epidemiología y Salud Pública (CIBERESP), Barcelona, Spain; 5 Institut d’Investigació Biomèdica (IIB Sant Pau), Carrer de Roc Boronat, 81-95, 08005 Barcelona, Spain

**Keywords:** Medical complexity, Comorbidity, Child, Health inequalities, Socioeconomic factors, Administrative data, Latent class analysis

## Abstract

**Background:**

Children with medical complexity (CMC) denotes the profile of a child with diverse acute and chronic conditions, making intensive use of the healthcare services and with special health and social needs. Previous studies show that CMC are also affected by the socioeconomic position (SEP) of their family. The aim of this study is to describe the pathologic patterns of CMC and their socioeconomic inequalities in order to better manage their needs, plan healthcare services accordingly, and improve the care models in place.

**Methods:**

Cross-sectional study with latent class analysis (LCA) of the CMC population under the age of 15 in Catalonia in 2016, using administrative data. LCA was used to define multimorbidity classes based on the presence/absence of 57 conditions. All individuals were assigned to a best-fit class. Each comorbidity class was described and its association with SEP tested. The Adjusted Morbidity Groups classification system (Catalan acronym GMA) was used to identify the CMC. The main outcome measures were SEP, GMA score, sex, and age distribution, in both populations (CMC and non-CMC) and in each of the classes identified.

**Results:**

71% of the CMC population had at least one parent with no employment or an annual income of less than €18,000. Four comorbidity classes were identified in the CMC: oncology (36.0%), neurodevelopment (13.7%), congenital and perinatal (19.8%), and respiratory (30.5%). SEP associations were: oncology OR 1.9 in boys and 2.0 in girls; neurodevelopment OR 2.3 in boys and 1.8 in girls; congenital and perinatal OR 1.7 in boys and 2.1 in girls; and respiratory OR 2.0 in boys and 2.0 in girls.

**Conclusions:**

Our findings show the existence of four different patterns of comorbidities in CMC and a significantly high proportion of lower SEP children in all classes. These results could benefit CMC management by creating more efficient multidisciplinary medical teams according to each comorbidity class and a holistic perspective taking into account its socioeconomic vulnerability.

## Background

Childhood is widely recognised as one of the population groups that warrants special care and attention, even more so when they suffer chronic comorbidities and severe limitations – known as children with medical complexity (CMC) [1], one of the most vulnerable populations. Studies differ regarding the prevalence of CMC status, ranging between 0.4% [2] and 0.7% [3] of total child population, although it is rising, given the continuous increase in their survival rates [4–8].

Children in this population group have complex acute and chronic conditions, numerous and varied comorbidities (from cerebral palsy to congenital heart defects or cancer), a broad range of mental health and psychosocial needs, major functional limitations, and a higher rate of mortality [1, 2, 6, 9]. They are under the continuous care of multiple paediatric specialists and require access to specialised care units^6^. As such, the CMC status indicates a child with intensive use of healthcare services and special health and social needs [10, 11]. Although they represent a small proportion of the population, CMC account for a substantial proportion of healthcare costs [3], and impact on other externalities such as family resources, psychological stress, and social exclusion [12–15].

Previous studies have examined socioeconomic position (SEP) [16] and ethnic inequalities [17] in CMC [11], and found that the prevalence of life-limiting conditions is higher in non-white and the most deprived CMC in England [7]. In Catalonia, low-SEP children are twice as likely to be CMC than those at the highest socioeconomic level [18, 19]. However, a study conducted in Wales did not find an association between mortality rates in paediatric intensive care units and SEP, despite noting an increase in the most vulnerable categories, especially among some ethnic groups [17].

With few exceptions [2], the research to date has focused on CMC with diseases within intensive care units, where accessible data elements are often restricted to the hospital setting [4, 6–8, 20, 21]. A wider approach is essential in order to obtain evidence that can guide the coordination of healthcare resources targeted to the different CMC profiles more efficiently [1].

The aim of this study is to describe more accurately te pathologic patterns of CMC (by clustering health diseases [22, 23]) and their socioeconomic inequalities in order to better manage better their needs, plan healthcare services accordingly, and improve the care models in place.

## Methods

### Study population

We selected the CMC individuals from the population of Catalonia under the age of 15 in 2016 (1,189,325). CMC individuals were identified by GMA score [24], a risk tool which classifies each individual into a health status and a severity level group, using administrative data. The higher the GMA score, the greater the individual’s medical complexity. To construct GMA score, comorbidity and severity information is gathered automatically from the Catalan Health Surveillance System (CHSS) database, for present and previous years. Each person in contact with the Catalan health system has a GMA score; this scoring is used to stratify the population for the purposes of health planning [24, 25]. It is more accurate and yields less variability than other health risk tools, such as Clinical Risk Group (CRG) [25], and has been approved by the World Health Organisation [26] (see Additional file 1 for further details). According to the GMA percentiles, the population is distributed in relation to clinical complexity (P_50_ very low risk, P_75_ low risk, P_85_ moderate risk, P_90_ high risk, P_99_ very high risk, P_99,5_ extreme risk).

We identified the CMC population based on the children included in the top 0.5% of GMA scores (P_99,5_). This criteria was applied since: 1) stratification tools have proven useful in determining CMC [2, 3, 21, 27, 28]; 2) this is the highest level of complexity indicated by the GMA; 3) previous studies in Catalonia have found that 0.3% of the population were CMC [18]; and 4) concordance with the prevalence of CMC in other population studies [2, 3]. As a comparative group, we used the remainder of the child population (non-CMC), representing 99.5% of that population.

### Data

We used two main sources of data: 1) The central registry of insured persons was used to obtain the reference population (as of January 1, 2016) based on their income level, employment status, and Social Security benefits; 2) the CHSS database includes detailed information on sociodemographic characteristics and medical diagnoses at an individual level in all contacts with primary care, emergency care, mental healthcare, long-term care services. All the historical comorbidities are updated if they are relevant, and it includes the whole population of Catalonia, since all citizens are granted universal health coverage.

### Variables

The main outcome variable is the different classes obtained by grouping patients with similar patterns of comorbidity. Comorbidities for all CMC were gathered from all the diagnoses registered and updated from 2014 to 2016. Diagnoses were coded using the Agency for Healthcare Research and Quality’s Clinical Classification Software (CCS) [29]. From a list of 184 relevant CCS, we grouped them into disease categories in order to facilitate information management. For each different CCS, it was only counted once in each individual. To obtain consistent and clinically relevant patterns of association, and to avoid spurious relationships that could bias the results, we considered only diagnosis categories with a prevalence of > 1%. Finally, 57 disease categories were included, covering 90.6% of all diseases (see Additional file 2).

For the exposure variable, the SEP of each child was measured based on economic information relating to one of their parents or guardians, including: employment status, individual income, and the receipt of welfare assistance. SEP was grouped into three categories: low (no member of the household employed or in receipt of welfare support from the government, and an income <€18,000/year, considered at risk of poverty [30]); middle (guardian employed with an income <€18,000); and high (guardian employment, with income >€18,000).

Age was categorised based on clinical criteria for children’s growth (0–1, 2–4, 5–11, 12–14) and used as the covariate, and sex was used as the stratification variable.

### Statistical analysis

A descriptive analysis of both the CMC and non-CMC populations was carried out. Bivariate analysis was conducted to determine differences between CMC and non-CMC groups according to sex, age, SEP, and GMA; proportion tests and Chi-square tests (for categorical variables) and a T-test or Mann–Whitney U (for continous variables) test were carried out depending on variable distribution.

Next, we used latent class analysis (LCA) [31] to classify CMC into patterns of comorbidity according to their distribution of disease categories. The objective of LCA is to classify individuals from an apparently heterogeneous population into more homogenous subgroups (latent classes) based on a number of observed indicators, in this case, the 57 disease categories.

To determine the optimal number of latent classes to fit the data, we used the Bayesian Information Criterion (BIC) and Akaike’s Information Criterion (AIC). An overall χ2 statistic was used to assess the model [32]. We compared candidate models and applied substantive interpretability and clinical judgement (i.e., do the classes defined by a given model possess a clinical significance or meaning?). After selecting a latent class model, we assigned each participant to his or her ‘best-fit’ class, meaning the class for which the participant had the highest computed probability of membership.

Subsequently we describle age, SEP, and GMA distribution in each class found in the LCA analysis by sex. Bivarate analysis was conducted to determine differences between boys and girls – a proportion test and Chi-square test, and T-tests or Mann-Whitney U tests were carried out. Finally, regression logistic models were used to examine the relationship between class membership and SEP with confidence intervals at 95% (CI95%) and their *p*-values.

All the analyses were carried out for boys and girls, separately. For all tests, the accepted significance level was 0.05 and adjusted by age. LCA was performed using the poLCA package [33] and R statistical software, version 3.3.1 [34], for conducting all analyses.

## Results

### Characteristics of the CMC population

The main characteristics of the CMC (0.5%) and non-CMC (99.5%) populations are described in Table 1. Both populations contained a higher proportion of boys (CMC 58.5% versus non-CMC 51.1%) than girls.

**Table 1:** Characteristics of children under 15 by population (CMC^a^ and non-CMC ^b^) and sex in Catalonia, 2016

	Boys					Girls				
	CMC		Non-CMC			CMC		Non-CMC		
	N	%	N	%	***P*** Value^**d**^	N	%	N	%	***P*** Value^**d**^
**Frequency**	3480	58.4	609,015	51.4	**<.001**	2470	41.6	574,360	48.6	**<.001**
**Age (years)**										
< 2	878	25.2	70,465	11.6	**<.001**	604	24.4	66,591	11.6	**<.001**
2 to 4	916	26.3	113,705	18.7		636	25.8	107,284	18.7	
5 to 11	1256	36.1	303,127	49.8		848	34.3	285,247	49.6	
12 to 14	430	12.4	121,718	19.9		382	15.5	115,238	20.1	
**SEP**										
Low	440	12.7	53,360	8.8	**<.001**	317	12.9	50,261	8.8	**<.001**
Middle	2030	58.5	321,762	52.8		1428	58.1	303,039	52.8	
High	1001	28.8	233,197	38.4		713	29.0	220,437	38.4	
**GMA**^**c**^**(score)**	16.7	(15.1–20.5)	2.3	(0.8–4.1)	**<.001**	16.7	(15.0–20.0)	2.1	(0.7–3.8)	**<.001**

Note: *GMA* Morbidity Adjusted Group, *SEP* Socioeconomic Position. Low (none member of the household employed, receiving welfare support from the government and an income < 18,000€/year), Middle (employed and an income < 18,000€/year), High (employed and an income > 18,000€/year)

^a^Children Medically Complex population = top 0.5% of GMA score of all entire population under 15

^b^Non Children Medically Complex population = 99.5% bottom of GMA score of all entire population under 15

Values are absolute numbers (percentages) for categorical variables. ^c^Median (IQR)

^d^*P* Value χ^2^ test for categorical variables and Mann-Whitney U-test for continuous variables. Differences between CMC and Non-CMC populations according to sex groups. α = 0.005

Almost a quarter of CMC were in the two first years of life (25.2% boys and 24.4% girls); compared with the non-CMC population; this rate was 2.2 times higher in boys and 2.1 times higher in girls. Approximately 50% of CMC of both sexes were aged under five, compared with around 30% of non-CMC; the rate was 69.7% higher in boys and 65.7% higher in girls (Table 1).

In terms of SEP, 71.1% of CMC (6.6% of non-CMC) had at least one parent with an annual income of less than €18,000 (low and middle SEP). Low SEP had a prevalence of 12.8% in the CMC group (12.7% in boys and 12.9% in girls) compared to 8.8% in non-CMC in both boys and girls; it is 44.5% higher in boys and 46.6% higher in girls in CMC than in the non-CMC group.

### Comorbidity classes of CMC

The smallest BIC and AIC values were obtained for the 4-class and 5-class candidate models. (see Additional file 3 for statistical values); after applying clinical criteria and χ2 value, we selected the 4-class model. The four classes were labelled based on which conditions exhibited more prevalence: oncology, neurodevelopment, congenital and perinatal, and respiratory.

Prevalences of all disease categories in each class are summarised in Additional file 4. Upper respiratory disease, infection, gastrointestinal disorders, fractures and injuries, and ear, eye, and skin disorders were highly present in all classes.

**Fig. 1:**
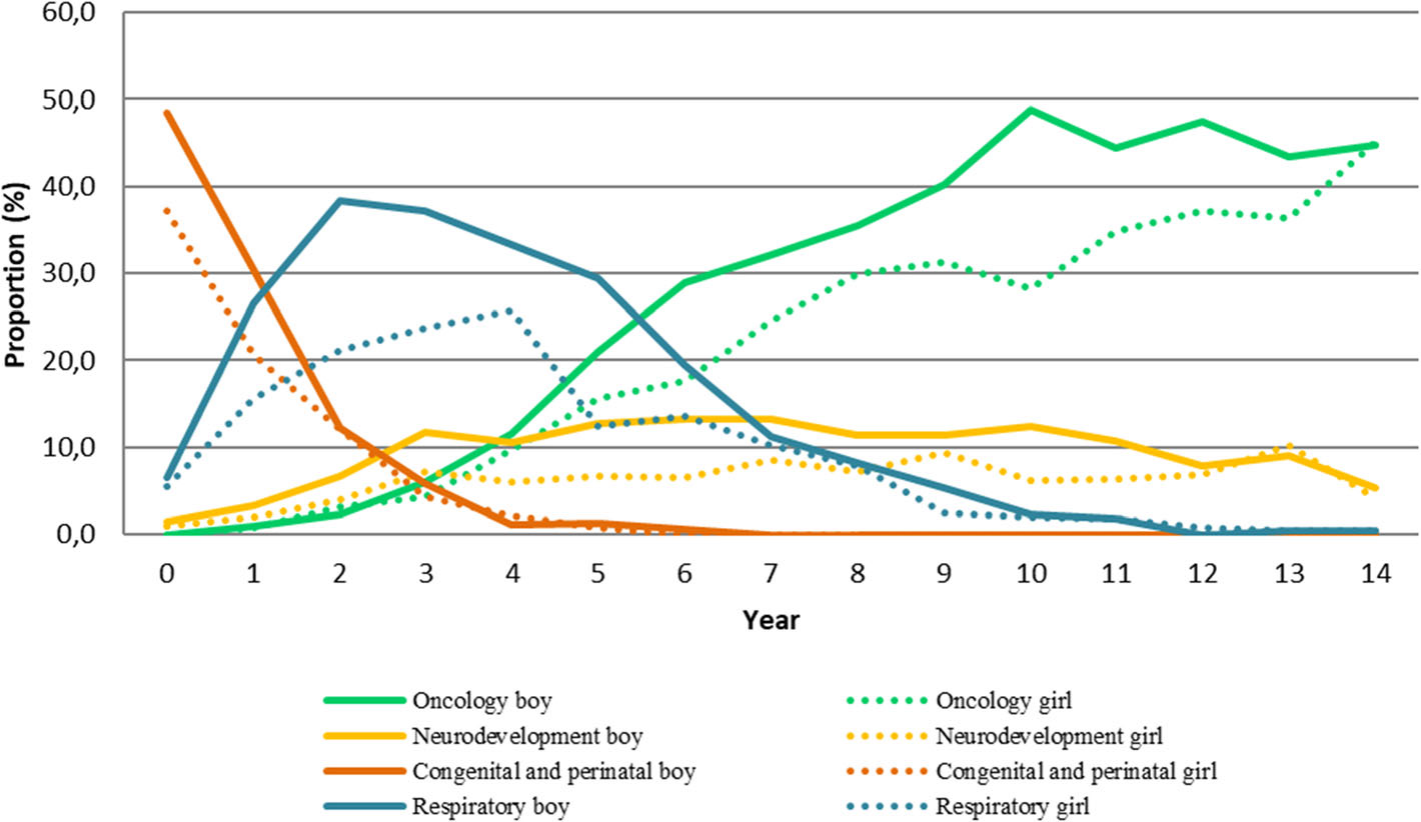
Proportion of comorbidity classes among CMC by age and sex, in Catalonia, 2016

**Fig. 2:**
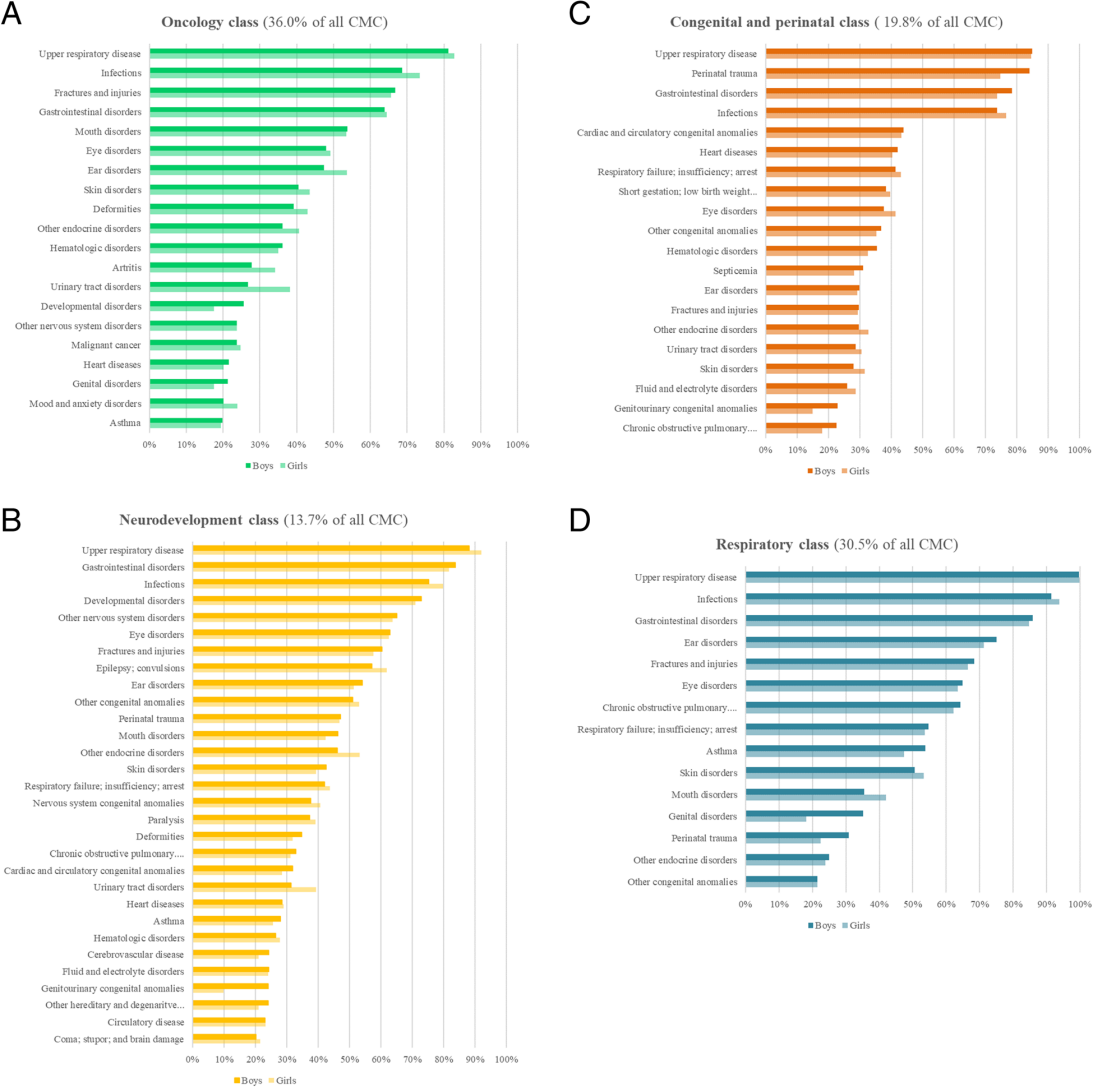
**a** Most prevalent diseases (> 20%) in the oncology class. **b** Most prevalent diseases (> 20%) in the neurodevelopment class. Abreviations: Chronic obstructive pulmonary disease and bronchiectasis. Other hereditary and degenerative nervous system conditions. **c** Most prevalent diseases (> 20%) in the congenital and perinatal class. Abreviations: Short gestation; low birth weight; and foetal growth retardation. Chronic obstructive pulmonary disease and bronchiectasis. **d** Most prevalent diseases (> 20%) in the respiratory class. Abreviations: Chronic obstructive pulmonary disease and bronchiectasis

The characteristics of the classes are summarised in Table 2 and their distribution during childhood is shown in Fig. 1. The SEP and age distribution of each obtained class is summarised in Table 2 and Fig. 1. Figures 2a,b,c,d display the most prevalent diseases (> 20%) in each of the four classes.

**Table 2:** Socioeconomic characteristics of each comorbidity cluster among the CMC and association between SEP by cluster, in Catalonia, 2016

	Oncology^**a**^					Neurodevelopment^**a**^					Congenital and perinatal^**a**^					Respiratory^**a**^				
	*N* = 2141 36.0%					*N* = 818 13.7%					*N* = 1177 19.8%					*N* = 1814 30.5%				
	Boys		Girls		***P*** Value	Boys		Girls		***P*** Value	Boys		Girls		***P*** Value	Boys		Girls		***P*** Value
	N	%	N	%		N	%	N	%		N	%	N	%		N	%	N	%	
Frequency	1200	56.1	941	44.0	0.004	501	61.3	317	38.8	0.083	668	56.8	509	43.3	0.182	1110	61.2	704	38.8	0.005
Ages (years)^b^																				
< 2	8	0.7	7	0.7	0.214^π^	38	7.6	23	7.3	0.126^π^	552	82.6	400	78.6	0.195^π^	280	25.2	174	24.7	0.569^π^
2 to 4	95	7.9	84	8.9		145	28.9	86	27.1		109	16.3	106	20.8		566	51.0	361	51.3	
5 to 11	729	60.8	529	56.2		258	51.5	151	47.6		7	1.1	3	0.6		262	23.6	165	23.4	
12 to 14	368	30.7	321	34.1		60	12.0	57	18.0		0	0.0	0	0.0		2	0.2	4	0.6	
SEP^b^																				
High	159	13.3	134	14.3	0.749^π^	69	13.9	40	12.8	0.297^π^	69	10.4	55	10.9	0.534^π^	143	12.9	88	12.5	0.9443^π^
Middle	664	55.4	507	54.2		299	60.2	176	56.2		408	61.3	323	63.7		658	59.3	422	60.1	
Low	375	31.3	295	31.5		129	26.0	97	31.0		189	28.4	129	25.4		308	27.8	192	27.4	
SEP OR (CI)^d^																				
High	1		1			1		1			1		1			1		1		
Middle	1.3 (1.2–1.5)		1.3 (1.1–1.5)			1.6 (1.3–2.0)		1.3 (1.0–1.7)			1.3 (1.1–1.6)		1.5 (1.3–1.9)			1.4 (1.2–1.6)		1.5 (1.2–1.7)		
Low	1.9 (1.6–2.3)		2.0 (1.7–2.5)			2.3 (1.7–3.1)		1.8 (1.2–2.6)			1.7 (1.3–2.3)		2.1 (1.5–2.8)			2.0 (1.7–2.5)		2.0 (1.6–2.6)		
GMA^c^ (score)	16.8 (15.2, 20.6)		16.9 (15.1–20-6)		0.555^γ^	20.9 (16.8, 26.2)		19.5 (16.2–26.5)		0.182^γ^	17.6 (15.4, 21.5)		16.9 (15.2, 20.2)		0.058^γ^	15.8 (14.7, 17.4)		15.8 (14.7, 17.4)		0.821^γ^

Note: *GMA* Morbidity Adjusted Group, *SEP* Socioeconomic Position. Low (no member of the household employed, receiving welfare support from the government, and an income < 18,000€/year), middle (employed and an income < 18,000€/year), high (employed and an income > 18,000€/year)

^a^ Proportion of each comorbidity class (%): (num of individuals in class/num individuals in all CMC)*100

^b^Values are absolute numbers and percentages in each class for categorical variables. ^c^Median (IQR) for continuous variables

*P* Value for categorical variables: π χ^2^ Test. For continuous variables: γ Mann-Whitney U-test. α = 0.005

^d^Odds Ratio, adjusted by age. CI, 95% confidence intervals of the odds ratio

Oncology class (Fig. 2a): includes 2141 children (36.0% of the CMC). Distribution was highest up to five years. There was a high proportion of oncological and related diseases: malignant cancer (23.7% boys, 24.7% girls), leukaemia (12.3% boys, 10.8% girls), cancer of the brain and nervous system (6.4% boys, 7.1% girls), and haematological disorders (36.1% boys, 35.0% girls).

Neurodevelopment class (Fig. 2b): includes 818 children (13.7% of the CMC). Distribution is fairly constant from 3 years and aupwards. Among the most prevalent diseases were developmental disorders (72.9% boys, 71.0% girls), other nervous system disorders (65.1% boys, 63.7% girls), epilepsy and convulsions (57.1% boys and 61.8% girls), and paralysis (37.7% boys and 39.1% girls).

Congenital and perinatal class (Fig. 2c): includes 1177 children (19.8% of the CMC). Distribution is mainly up to 4 years old. Perinatal trauma (84.1% boys, 74.7% girls), cardiac and circulatory congenital anomalies (43.9% boys, 43.2% girls), short gestation, low birth weight, and foetal growth retardation (38.3% boys, 39.7% girls), and other congenital anomalies (36.8% boys, 35.2% girls) were the most frequent diseases.

Respiratory class (Fig. 2d): included 1814 (30.5% of the CMC). It shows an accumulation of individuals aged between years 1 and 6. The most prominent diseases were chronic obstructive pulmonary disease and bronchiectasis (64.2% boys, 62.1% girls), respiratory failure, insufficiency and arrest (54.7% boys, 53.6% girls), and asthma (53.7% boys, 47.4% girls).

### SEP inequalities

**Fig. 3:**
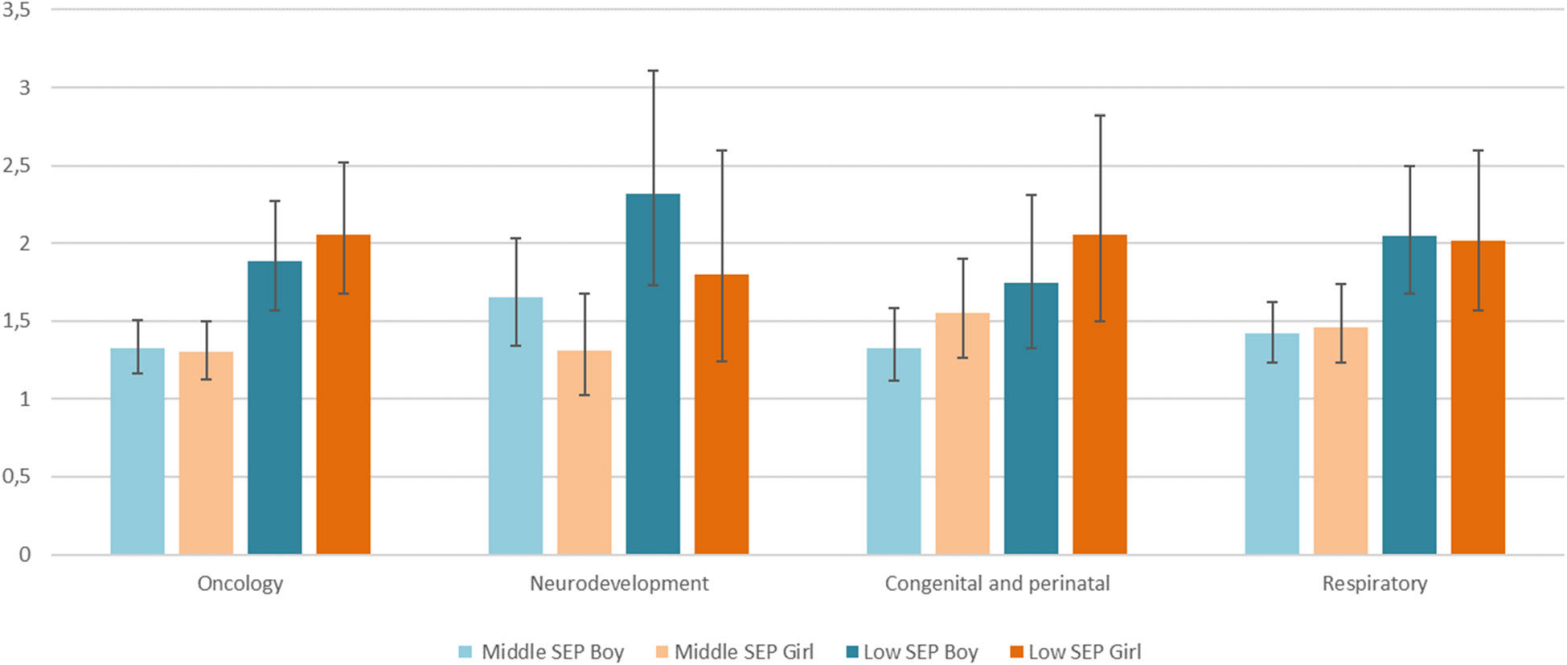
Odds ratio between socioeconomic position (SEP) and each comorbidity class among CMC by sex. Catalonia, 2016.*. *Models were adjusted by age. Odds ratio and 95% Confidence Interval. High SEP was the reference category

SEP inequalities in the four clusters are displayed in Table 2 and Fig. 3. There were SEP inequalities in all clusters, for both sexes. From higher to lower OR in one of both sexes, neurodevelopment class showed an association with low SEP ([OR, 2.3; CI95%, 1.7–3.1 in boys] and [OR, 1.8; CI95%, 1.2–2.6 in girls]) compared to the high SEP category, congenital and perinatal class ([OR, 2.1; CI95%, 1.5–2.8 in girls]) and [OR, 1.7; CI95%, 1.3–2.3 in boys]), followed by respiratory class ([OR, 2.0; CI95%, 1.6–2.6 in girls] and [OR, 2.0; CI95%, 1.7–2.5 in boys]), and finally the oncology class ([OR, 2.0; CI95%, 1.7–2.5 in girls] and [OR, 1.9; CI95%, 1.6–2.3 in boys]).

## Discussion

Four different comorbidity classes among the CMC were identified. All of them showed SEP inequalities, therefore the more disadvantaged children represent a higher proportion of the CMC group. In both populations (CMC and non-CMC) and sexes, > 60% of children were low and middle SEP. This finding highlights the fact that children are subject to inequalities from the very beginning of their lives [35].

In this study, all CMC classes shared common diseases – specifically gastrointestinal disorders, respiratory diseases, and trauma – as in other population studies [20, 21]. These diseases account for the major causes of hospitalisation rates together with congenital anomalies, and cardiovascular and oncological diseases [7, 21].

All classes had a higher proportion of boys, up to 56%. This result is consistent with the highest vulnerability in boys aged up to five years; male foetuses mature slower than female foetuses do and, after birth, males experience more perinatal issues [36]. This also coincides with the maximum prevalence of the congenital and perinatal, and respiratory classes (99.1 and 76.1%, respectively).

The oncology class contained 36.0% of all CMC and was predominated by individuals aged up to 5 years. Their characteristics were more heterogeneous and showed a higher comorbidity profile. Although this class included almost all the individuals with malignancies, individuals with mental health or endocrine disorders were also highly represented. This pattern is consistent with other studies that emphasise that when a CMC matures he or she could develop more than one comorbidity as a result of their main pathology [20], and often this new comorbidities are related to mental disorders [37].

The neurodevelopment class includes two related types of diseases: nervous system disorders such as paralysis and epilepsy, and congenital, perinatal, and degenerative anomalies. Their prevalence remains steady as the child grows older; they have a chronic, cumulative profile due to the difficulty of healing, and they may be precursors of future complications in other systems [38]. The aetiology of nervous system anomalies may be related to SEP inequalities, such as exposure to certain environmental factors [39], maternal stress during pregnancy, or adverse gestational and delivery outcomes [40, 41]. All these events occur in the prenatal and perinatal period but their impact may emerge at a later stage. This class showed the highest median GMA, since the prognosis and development of the pathology entail a high risk and large use of healthcare resources.

The congenital and perinatal disease class comprises mainly adverse birth outcomes and congenital anomalies in diverse body systems, especially heart defects in concordance with the principal incidence of congenital anomalies in other populations [42]. The maximum prevalance of the congenital and perinatal class was observed in the first two years of life, due to the congenital aetiology. In this short time, SEP influences the child mainly via the mother: maternal behaviour during pregnancy has been identified as a risk factor [43–45]. It should also be noted that advances in perinatal care have increased the likelihood of survival for extremely preterm infants, who are mostly included in this class.

The respiratory class includes mainly pulmonary diseases. In accordance with the natural development of most respiratory diseases, its prevalence was highest in the mid-age range, and it accounted for 30% of all the CMC. Risk factors known to be related to SEP inequalities in this class are: exposure to air pollution [46], in utero exposure to tobacco [47], maternal stress [45], and low weight at birth and prematurity [48].

The age distribution of each class showed the ages of maximum expression of each of the patterns (see Fig. 1). It should be noted that diseases are not static, and prognosis may mean that individuals move from class to class.

All CMC classes showed SEP inequalities, thus corroborating the previous analyses carried out in Catalonia [18, 19] and elsewhere [7, 16]. SEP inequalities were similar across the four classes, which highlights the lack of economic support in accessing the best development and care that these children and their family experience. Our study denotes that family’s SEP is related to CMC. This fact could impact on their development and hindering these children from achieving their potential. Having to care for CMC may also further negatively affect families’ economic position and health [12, 14, 15] and may, in turn, affect the CMC. In contrast, families with more economic resources are able to provide more active stimulation, alternative treatments, and an environment that is safer and more conducive to maintaining good health in childhood. This phenomenon has been termed the “buffering effect of income” in chronic conditions [38]. On the other hand, these results denote that inequities are already established in the first years of life, suggesting that there is a pattern of causality as indicated by different studies of highly disabling diseases [16]. The idea that, since conception, SEP inequalities are an important factor determining the developmental origin of different diseases, is increasingly gaining more evidence, establishing that the mother and the family environment are key to the production of disease [49]. Ensuring this pattern in all diseases is challenging, but our results, especially in regard to the youngest CMC, cannot be explained by reverse causality alone. This indicates that the issue warrants further research. According to different experts on CMC [11], other social determinants of health such as ethnicity, immigration status, or geographical isolation, influence CMC’s health outcomes. Our data does not allow for deeper insights on ethnicity; this should be explored further in future research as some studies have identified it as a factor having more influence than SEP [7, 17].

### Study strengths and limitations

Identifying CMC at a population level is not straightforward. There is no specific agreed criteria for definingthe CMC population, and all of the proposed criteria have present limitations. The GMA, like other classification systems, was originally created for the whole population (children and adults). Nevertheless, Clinical Risk Groups based on the same principle and have been successfully used to identify CMC populations [2, 3, 50, 51]. Furthermore, clinical diagnoses across the historical healthcare contacts have been considered as it is recommend [28], providing a more realistic approach to the health status of the children.

Because of the limited data available on income, we were unable to obtain a more detailed segmentation of the SEP variable. This was especially true in the case of the high SEP category, which included a wide range of income levels. Further segmentation of this category would have given a more accurate approximation of the SEP gradient. However, parental income is the SEP indicator that most directly measures the family’s material resources. With other indicators, such as labour, income has a ‘dose-response’ association with health [52]. Some studies have used maternal education or an ecological deprivation index as a proxy for SEP [7, 16, 17]; the present study goes further by using population-based individual income data, which is more directly related to the material resources.

The health status data is based on the use of public healthcare resources, since data from private healthcare providers was not available. Even so, the bias is presumably very low, as CMC patients require highly specialised care and, for this reason, are mainly treated in the public healthcare system.

The main strength of this population-based study is the use of robust individual administrative data, like similar studies and databases [2, 6, 7]. Another advantage is that it includes all the children in Catalonia and thus provides a realistic view of the current health status of the population beyond hospital-based care. Hospital-based studies do not address outpatient utilisation of services and do not reflect the highest-risk patients, as they are treated in specialised units; meanwhile, our study sheds light on all the comorbidities adjacent to the CMC population with a more chronic profile.

## Conclusion

Our findings have demonstrated the existence of different patterns of comorbidities in CMC and a high proportion of lower socioeconomic children in all classes. This result could benefit CMC management by enabling the creation of more efficient multidisciplinary teams according to each comorbidity class and informing a holistic perspective taking into account the socioeconomic vulnerability this population faces.

Daily life for CMC and their families is not only complex from the perspective of healthcare; every area of life is complex. Child health and family health are two sides of the same coin. Introducing policies to support both their health and financial situation will have implications beyond children’s health itself.

## Supplementary information

**Additional file 1.** Adjusted morbidity groups. Description of data: Details of the adjusted morbidity groups construction.

**Additional file 2.** List of the Clinical Classifications Software (CCS) for ICD-9-MC. included in each disease category (covering 90.6% of all the disease events). Description of data: Clinical codes included in each disease category.

**Additional file 3.** LCA statistics. Description of data: LCA statistics for all the models used. It included Chisq Chi_square goodness of fit, Bayesian Information Criterion, Akaike’s Information Criterion, Log_likelihood, Consistent Alkaike’s Information Criterion and Likelihood Ratio chi-square.

**Additional file 4.** Prevalences of all disease categories by sex for each comorbidity class among the CMC in Catalonia, 2016. Description of data: Prevalences of all the disease categories for each of the comorbidity classes obtained in the LCA. This data shows the frequencies and percentages of each disease category by sex for each of the classes obtained.

## Data Availability

The data that support the findings of this study are not publicly available due to the presence of personal information that could compromise research participants’ privacy. The anonymised and unidentified data will be accessible to the research staff of the research centres accredited by the Research Centres of Catalonia (CERCA) institution, SISCAT agents, and public university research centres, as well as the same health administration.

## Abbreviations

CMC: Children with medical complexity
SEP: Socioeconomic position
Catalan acronym GMA: The Adjusted Morbidity Groups
CHSS: Catalan Health Surveillance System
CRG: Clinical Risk Group
CCS: Clinical Classification Software
LCA: Latent Class Analysis
BIC: Bayesian Information Criterion
AIC: Akaike’s Information Criterion
