# Supplementary material for: Comorbidity patterns and socioeconomic inequalities in children under 15 with medical complexity: a population-based study
Source: BMC Pediatr. 2020 Jul 30;20:358. doi: 10.1186/s12887-020-02253-z (PMC7391621; doi:10.1186/s12887-020-02253-z)
Supplement: Supplementary file 1 — Additional file 1. Adjusted morbidity groups. Description of data: Details of the adjusted morbidity groups construction. [file 12887_2020_2253_MOESM1_ESM.pdf]

Additional file 1. Adjusted Morbidity Groups

To be able to respond to the healthcare needs of the population and to manage resources efficiently, healthcare systems need to know the care needs of users based on their health problems. In order to keep track of this information, there is a range of tools on the market that allow people to be classified into various categories, depending on the health problems they suffer from and the severity of those problems. This classification determines the need for health resources in each group. The most well-known of these tools are the Clinical Risk Group (CRG) model, developed by 3M, and the Adjusted Clinical Groups (ACG) model, by Johns Hopkins University, Baltimore (United States of America). These models, used worldwide, calculate clinical complexity using care and economic criteria, but are based on the American healthcare reality.

A new clinical grouping system called Adjusted Morbidity Groups (GMA) and developed by David Monterde, responsible for the Statistical Office of the Information Systems Area of the Management Directorate of the Institut Català de la Health (ICS), together with Emili Vela and Montse Clteries, technicians of the Demand Analysis Division and Activity Activity and Quality of the Catalan Health Service (CatSalut), overcomes the limitations of the previous models in the context of the Catalan health system.

Model of success

GMA has proven to be a more effective predictive grouping tool than CRG in the Catalan healthcare system, and also allows each individual to be assigned a unique value of complexity, which is not possible in the previous models. The success of the GMA has led the Ministry of Health to consider using it as a reference grouping system for the entire National Health System (Catalan acronym SNS). It has also been approved as a stratification risk tool for the World Health Organisation.

In 2014, the Community of Madrid launched a pilot study to validate GMA in their health contetx. The results of this study were published in the digital edition of Atención Primaria in September 2016. In 2015, as part of an agreement with the Ministry, GMA was implanted in 13 communities: Andalusia, Aragon, the Balearic Islands, the Canary Islands, Cantabria, Castilla La Mancha, Castile León, the Valencian Community, Extremadura, Galicia, Madrid, Murcia, and Navarra. As a result of this agreement, there are currently 38 million people in Spain who have been grouped according to GMA.

Three levels

GMA uses three levels of information. The first level is the classification of the population into unique morbidity groups that are simultaneously divided into different levels of complexity. In total, the Catalan model identifies seven groups: healthy population; pregnancy and childbirth; patients with acute disease; patients with chronic disease in one system (for example, diabetics); patients with chronic disease in two or three systems (for example, people with diabetes with heart failure and kidney failure); patients with chronic disease in four or more systems (diabetic people with heart failure, kidney failure and osteoarthritis); and patients with active neoplasia. Each of these groups is divided into five levels of severity, except for the healthy group, which has only one level. Combining morbidity and severity, there are a total of 31 groups.

The novelty of the model lies in the second level of information, in which a unique value of complexity is assigned to each individual. This value reflects the healthcare needs that people may have, based on their health problems. In particular, it takes into account the factors associated with multimorbidity (that is, the joint effects that an individual has on different diseases), a situation that is more a norm than an exception among chronic patients.

The third level, derived from the previous one, involves the identification of people with certain relevant health problems with a clinical label. A total of 80 conditions are identified at this level (among which are: diabetes II, COPD, neoplasms, high blood pressure, arthritis, and depression). Clinical etiquette allows for better follow-up of patients with more complex care needs.

Flexibility

The head of the ICS Statistics Office, Monterde, emphasises that, "the main advantage of GMA is that they are flexible; we can adapt them to the clinical and technical needs as appropriate." One of the things that has most appreciated is precisely this flexibility, which allows the model to be adapted to the reality of each region by developing new predictive models. However, this task will be completed at a later point. Currently, the implementation of GMA in the SNS is based on the data collected in the Catalan healthcare system.

Another benefit of implementing GMA as a clinical grouping system is that it saves on the costs of licences for other tools, such as CRG or ACG. The adoption of GMA in the SNS has led Catalan healthcare companies to earn more than €150,000 and save more than €100,000 per year oinICS licences for CRG.

Functionality of morbidity groups

Population stratification and identification of the population at risk

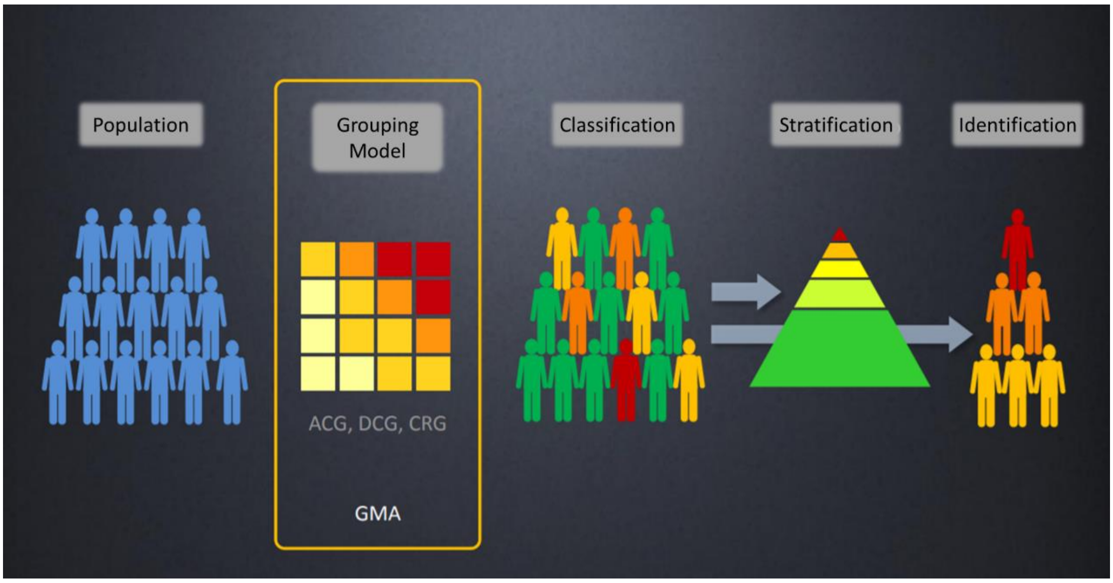

Architecture of GMA

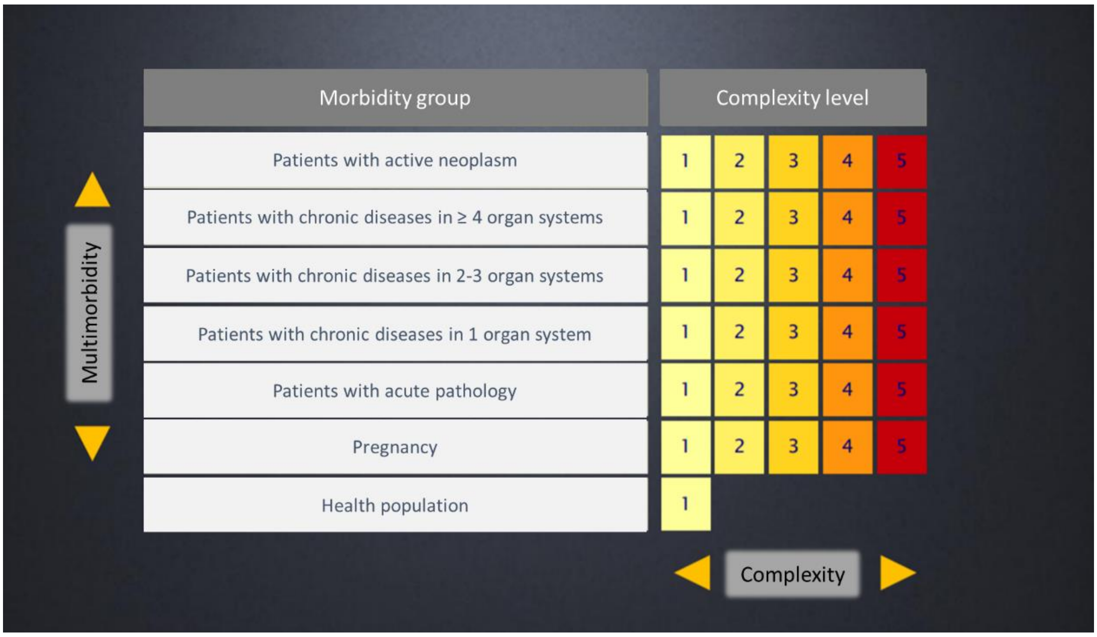

Stratification based on mortality according to GMA for the Catalonian population in 2012

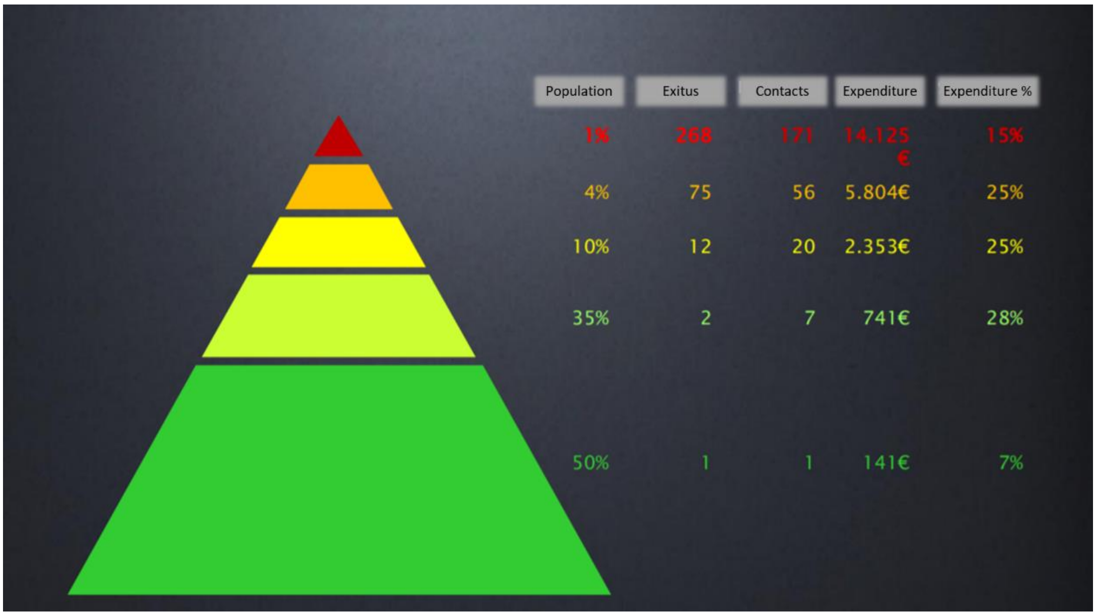

References

Monterde D, Vela E, Clèries M. Los grupos de morbilidad ajustados: nuevo agrupador de morbilidad poblacional de utilidad en el ámbito de la atención primaria. Aten Primaria. 2016;48(10):674–82.

Dueñas-Espín I, Vela E, Pauws S, Bescos C, Cano I, Cleries M, et al. Proposals for enhanced health risk assessment and stratification in an integrated care scenario. BMJ Open. 2016;6(4):e010301.

Cerezo J, Arias C. Population stratification: A fundamental instrument used for population health management in Spain. Good Practices Brief WHO.
