# Supplementary material for: Comorbidity patterns and socioeconomic inequalities in children under 15 with medical complexity: a population-based study
Source: BMC Pediatr. 2020 Jul 30;20:358. doi: 10.1186/s12887-020-02253-z (PMC7391621; doi:10.1186/s12887-020-02253-z)
Supplement: Supplementary file 2 — Additional file 2. List of the Clinical Classifications Software (CCS) for ICD-9-MC. included in each disease category (covering 90.6% of all the disease events). Description of data: Clinical codes included in each disease category. [file 12887_2020_2253_MOESM2_ESM.pdf]

Additional file 2. List of the Clinical Classifications Software (CCS) for ICD-9-MC included in each disease category (covering the 90.6% of all the diseases events).

Diseases categories	CCS	CCS Description
Septicemia (except in labor)	2	Septicemia (except in labor)
Infections	3	Bacterial infection; unspecified site
	4	Mycoses
	8	Other infections; including parasitic
	7	Viral infection
	123	Influenza
Hepatitis	6	Hepatitis
Malignant cancer	11	Cancer of head and neck
	12	Cancer of esophagus
	13	Cancer of stomach
	14	Cancer of colon
	15	Cancer of rectum and anus
	16	Cancer of liver and intrahepatic bile duct
	17	Cancer of pancreas
	18	Cancer of other GI organs; peritoneum
	19	Cancer of bronchus; lung
	20	Cancer; other respiratory and intrathoracic
	21	Cancer of bone and connective tissue
	22	Melanomas of skin
	23	Other non-epithelial cancer of skin
	24	Cancer of breast
	25	Cancer of uterus
	26	Cancer of cervix
	27	Cancer of ovary
	28	Cancer of other female genital organs
	29	Cancer of prostate
	30	Cancer of testis
	31	Cancer of other male genital organs
	32	Cancer of bladder
	33	Cancer of kidney and renal pelvis
	34	Cancer of other urinary organs
	36	Cancer of thyroid
	37	Hodgkin`s disease
	40	Multiple myeloma
	41	Cancer; other and unspecified primary
	42	Secondary malignancies
	43	Malignant neoplasm without specification of site
	44	Neoplasms of unspecified nature or uncertain behavior
	45	Maintenance chemotherapy; radiotherapy
Cancer of brain and nervous system	35	Cancer of brain and nervous system
Non-Hodgkin`s lymphoma	38	Non-Hodgkin`s lymphoma
Leukemias	39	Leukemias
Benign neoplasm	46	Benign neoplasm of uterus
	47	Other and unspecified benign neoplasm
Thyroid disorders	48	Thyroid disorders
Diabetis mellitus	49	Diabetes mellitus without complication
	50	Diabetes mellitus with complications
Other endocrine disorders	51	Other endocrine disorders

	58	Other nutritional; endocrine; and metabolic disorders
Nutritional deficiencies	52	Nutritional deficiencies
Disorders of lipid metabolism	53	Disorders of lipid metabolism
Fluid and electrolyte disorders	55	Fluid and electrolyte disorders
Immunity disorders	57	Immunity disorders
Hematologic disorders	61	Sickle cell anemia
	59	Deficiency and other anemia
	60	Acute posthemorrhagic anemia
	62	Coagulation and hemorrhagic disorders
	63	Diseases of white blood cells
	64	Other hematologic conditions
CNS infection	76	Meningitis (except that caused by tuberculosis or sexually transmitted disease)
	77	Encephalitis (except that caused by tuberculosis or sexually transmitted disease)
	78	Other CNS infection and poliomyelitis
Other hereditary and degenerative nervous system conditions	81	Other hereditary and degenerative nervous system conditions
Paralysis	82	Paralysis
Epilepsy; convulsions	83	Epilepsy; convulsions
Headache; including migraine	84	Headache; including migraine
Coma; stupor; and brain damage	85	Coma; stupor; and brain damage
Eye disorders	88	Glaucoma
	86	Cataract
	87	Retinal detachments; defects; vascular occlusion; and retinopathy
	89	Blindness and vision defects
	90	Inflammation; infection of eye (except that caused by tuberculosis or sexually tr
	91	Other eye disorders
Ear disorders	92	Otitis media and related conditions
	93	Conditions associated with dizziness or vertigo
	94	Other ear and sense organ disorders
Other nervous system disorders	95	Other nervous system disorders
Essential hypertension	98	Essential hypertension
	99	Hypertension with complications and secondary hypertension
Heart diseases	103	Pulmonary heart disease
	104	Other and ill-defined heart disease
	105	Conduction disorders
	106	Cardiac dysrhythmias
	107	Cardiac arrest and ventricular fibrillation
	108	Congestive heart failure; nonhypertensive
	96	Heart valve disorders
	97	Peri-; endo-; and myocarditis; cardiomyopathy (except that caused by tuberculosis
Cerebrovascular disease	109	Acute cerebrovascular disease
	111	Other and ill-defined cerebrovascular disease
	113	Late effects of cerebrovascular disease
	110	Occlusion or stenosis of precerebral arteries
Circulatory disease	119	Varicose veins of lower extremity
	120	Hemorrhoids
	117	Other circulatory disease
	118	Phlebitis; thrombophlebitis and thromboembolism
	121	Other diseases of veins and lymphatics

Lower respiratory disease	122	Pneumonia (except that caused by tuberculosis or sexually transmitted disease)
	133	Other lower respiratory disease
	125	Acute bronchitis
Upper respiratory disease	126	Other upper respiratory infections
	134	Other upper respiratory disease
	124	Acute and chronic tonsillitis
Chronic obstructive pulmonary disease and bronchiectasis	127	Chronic obstructive pulmonary disease and bronchiectasis
Asthma	128	Asthma
Aspiration pneumonitis; food/vomitus	129	Aspiration pneumonitis; food/vomitus
Pleurisy; pneumothorax; pulmonary collapse	130	Pleurisy; pneumothorax; pulmonary collapse
Respiratory failure; insufficiency; arrest (adult)	131	Respiratory failure; insufficiency; arrest (adult)
Mouth disorders	136	Disorders of teeth and jaw
	137	Diseases of mouth; excluding dental
Gastrointestinal disorders	138	Esophageal disorders
	140	Gastritis and duodenitis
	141	Other disorders of stomach and duodenum
	142	Appendicitis and other appendiceal conditions
	153	Gastrointestinal hemorrhage
	154	Noninfectious gastroenteritis
	155	Other gastrointestinal disorders
	135	Intestinal infection
	143	Abdominal hernia
	145	Intestinal obstruction without hernia
	147	Anal and rectal conditions
	148	Peritonitis and intestinal abscess
	144	Regional enteritis and ulcerative colitis
Digestive system disease	149	Biliary tract disease
	151	Other liver diseases
	152	Pancreatic disorders (not diabetes)
Renal diseases	156	Nephritis; nephrosis; renal sclerosis
	157	Acute and unspecified renal failure
	158	Chronic kidney disease
Urinary tract disorders	159	Urinary tract infections
	160	Calculus of urinary tract
	161	Other diseases of kidney and ureters
	163	Genitourinary symptoms and ill-defined conditions
Genital disorders	165	Inflammatory conditions of male genital organs
	166	Other male genital disorders
	168	Inflammatory diseases of female pelvic organs
	171	Menstrual disorders
	175	Other female genital disorders
Skin disorders	197	Skin and subcutaneous tissue infections
	198	Other inflammatory condition of skin
	199	Chronic ulcer of skin
	200	Other skin disorders
Artritis	201	Infective arthritis and osteomyelitis (except that caused by tuberculosis or sexually transmitted disease)
	202	Rheumatoid arthritis and related disease
	203	Osteoarthritis
	204	Other non-traumatic joint disorders

	205	Spondylosis; intervertebral disc disorders; other back problems
Deformities	208	Acquired foot deformities
	209	Other acquired deformities
	211	Other connective tissue disease
	212	Other bone disease and musculoskeletal deformities
Cardiac and circulatory congenital anomalies	213	Cardiac and circulatory congenital anomalies
Digestive congenital anomalies	214	Digestive congenital anomalies
Genitourinary congenital anomalies	215	Genitourinary congenital anomalies
Nervous system congenital anomalies	216	Nervous system congenital anomalies
Other congenital anomalies	217	Other congenital anomalies
Short gestation; low birth weight; and fetal growth retardation	219	Short gestation; low birth weight; and fetal growth retardation
Perinatal trauma	220	Intrauterine hypoxia and birth asphyxia
	221	Respiratory distress syndrome
	222	Hemolytic jaundice and perinatal jaundice
	223	Birth trauma
	224	Other perinatal conditions
Fractures and injuries	228	Skull and face fractures
	226	Fracture of neck of femur (hip)
	225	Joint disorders and dislocations; trauma-related
	229	Fracture of upper limb
	230	Fracture of lower limb
	231	Other fractures
	232	Sprains and strains
	233	Intracranial injury
	235	Open wounds of head; neck; and trunk
	236	Open wounds of extremities
	239	Superficial injury; contusion
	244	Other injuries and conditions due to external causes
	240	Burns
Mood and anxiety disorders	650	Adjustment disorders
	657	Mood disorders
	658	Personality disorders
	651	Anxiety disorders
	661	Substance-related disorders
Attention-deficit, conduct, and disruptive behavior disorders	652	Attention-deficit, conduct, and disruptive behavior disorders
Developmental disorders	654	Developmental disorders
	655	Disorders usually diagnosed in infancy, childhood, or adolescence
Miscellaneous mental health disorders	670	Miscellaneous mental health disorders
