# Supplementary material for: Comorbidity patterns and socioeconomic inequalities in children under 15 with medical complexity: a population-based study
Source: BMC Pediatr. 2020 Jul 30;20:358. doi: 10.1186/s12887-020-02253-z (PMC7391621; doi:10.1186/s12887-020-02253-z)
Supplement: Supplementary file 3 — Additional file 3. LCA statistics. Description of data: LCA statistics for all the models used. It included Chisq Chi_square goodness of fit, Bayesian Information Criterion, Akaike’s Information Criterion, Log_likelihood, Consistent Alkaike’s Information Criterion and Likelihood Ratio chi-square. [file 12887_2020_2253_MOESM3_ESM.pdf]

**Additional file 3. Statistics for each Latent Class Model performed.**

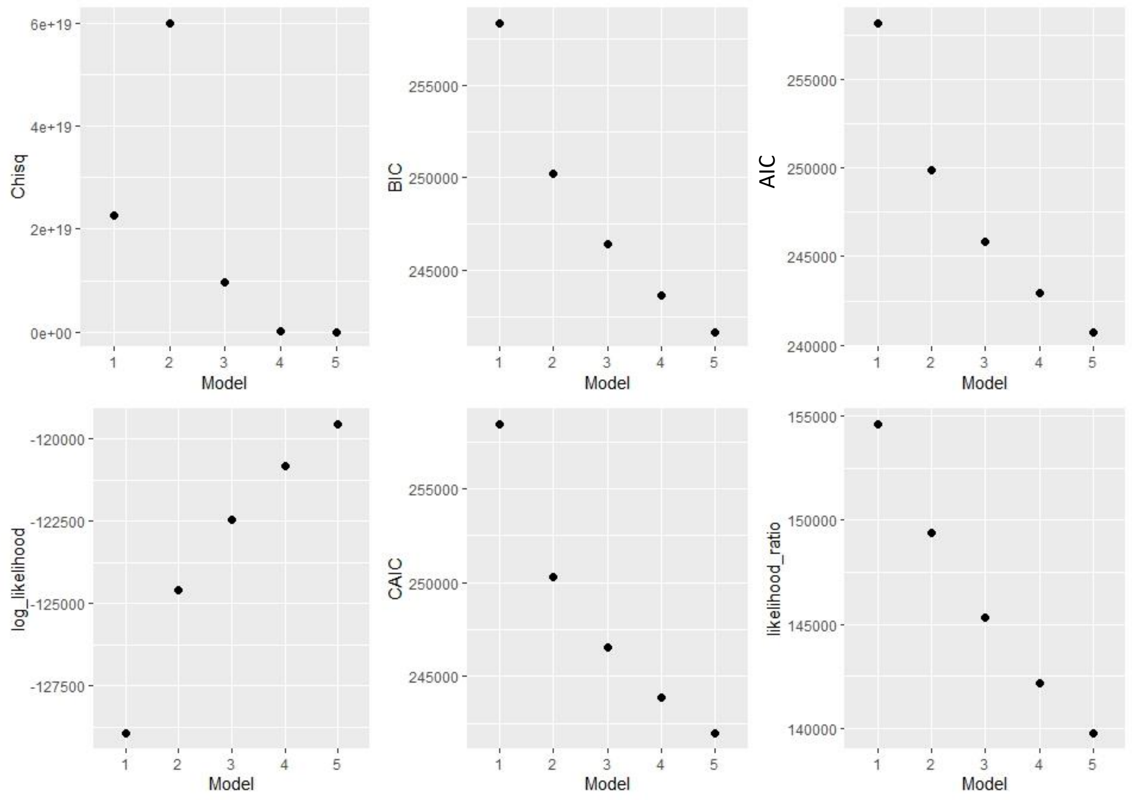


Chisq (Chi_square goodness of fit), BIC (Bayesian Information Criterion), AIC (Akaike's Information Criterion), Log_likelihood, CAIC (Consistent Alkaike's Information Criterion), Likelihood_ratio (Likelihood Ratio chi-square)
