# Supplementary material for: Comorbidity patterns and socioeconomic inequalities in children under 15 with medical complexity: a population-based study
Source: BMC Pediatr. 2020 Jul 30;20:358. doi: 10.1186/s12887-020-02253-z (PMC7391621; doi:10.1186/s12887-020-02253-z)
Supplement: Supplementary file 4 — Additional file 4. Prevalences of all disease categories by sex for each comorbidity class among the CMC in Catalonia, 2016. Description of data: Prevalences of all the disease categories for each of the comorbidity classes obtained in the LCA. This data shows the frequencies and percentages of each disease category by sex for each of the classes obtained. [file 12887_2020_2253_MOESM4_ESM.pdf]

## Additional file 3. Prevalences of all diseases categories by sex for each comorbidity class among the CMC in Catalonia, 2016.

| | Oncology class | | Neurodevelopment class | | Congenital and perinatal class | | Respiratory class | |
|---|---|---|---|---|---|---|---|---|
| | 2141 (36.0%) | | 818 (13.7%) | | 1177 (19.8%) | | 1814 (30.5%) | |
| **Diseases (N (%))** | **Boys** | **Girls** | **Boys** | **Girls** | **Boys** | **Girls** | **Boys** | **Girls** |
| Septicemia | 107 (8.9) | 74 (7.9) | 49 (9.8) | 31 (9.8) | 207 (31.0) | 143 (28.1) | 38 (3.4) | 20 (2.8) |
| Hepatitis | 35 (2.9) | 20 (2.1) | 5 (1.0) | 6 (1.9) | 4 (0.6) | 3 (0.6) | 8 (0.7) | 3 (0.4) |
| Cancer of brain and nervous system | 77 (6.4) | 67 (7.1) | 11 (2.2) | 7 (2.2) | 2 (0.3) | 3 (0.6) | 0 (0.0) | 0 (0.0) |
| Non-Hodgkin`s lymphoma | 53 (4.4) | 15 (1.6) | 0 (0.0) | 0 (0.0) | 0 (0.0) | 0 (0.0) | 0 (0.0) | 0 (0.0) |
| Leukemias | 148 (12.3) | 102 (10.8) | 0 (0.0) | 0 (0.0) | 8 (1.2) | 2 (0.4) | 0 (0.0) | 1 (0.1) |
| Thyroid disorders | 42 (3.5) | 39 (4.1) | 37 (7.4) | 23 (7.3) | 13 (1.9) | 10 (2.0) | 8 (0.7) | 3 (0.4) |
| Nutritional deficiencies | 67 (5.6) | 70 (7.4) | 76 (15.2) | 58 (18.3) | 65 (9.7) | 42 (8.3) | 22 (2.0) | 17 (2.4) |
| Disorders of lipid metabolism | 69 (5.8) | 48 (5.1) | 11 (2.2) | 8 (2.5) | 3 (0.4) | 1 (0.2) | 15 (1.4) | 12 (1.7) |
| Fluid and electrolyte disorders | 149 (12.4) | 136 (14.5) | 121 (24.2) | 76 (24.0) | 173 (25.9) | 146 (28.7) | 149 (13.4) | 90 (12.8) |
| Immunity disorders | 64 (5.3) | 67 (7.1) | 26 (5.2) | 10 (3.2) | 20 (3.0) | 10 (2.0) | 27 (2.4) | 22 (3.1) |
| Other hereditary and degenerative nervous system conditions | 78 (6.5) | 56 (6.0) | 120 (24.0) | 67 (21.1) | 22 (3.3) | 19 (3.7) | 10 (0.9) | 5 (0.7) |
| Paralysis | 46 (3.8) | 50 (5.3) | 187 (37.3) | 124 (39.1) | 4 (0.6) | 2 (0.4) | 7 (0.6) | 1 (0.1) |
| Epilepsy; convulsions | 114 (9.5) | 82 (8.7) | 286 (57.1) | 196 (61.8) | 55 (8.2) | 39 (7.7) | 75 (6.8) | 51 (7.2) |
| Headache; including migraine | 194 (16.2) | 198 (21.0) | 35 (7.0) | 18 (5.7) | 1 (0.1) | 0 (0.0) | 21 (1.9) | 17 (2.4) |
| Coma; stupor; and brain damage | 20 (1.7) | 21 (2.2) | 101 (20.2) | 68 (21.5) | 9 (1.3) | 15 (2.9) | 17 (1.5) | 4 (0.6) |
| Other nervous system disorders | 284 (23.7) | 224 (23.8) | 326 (65.1) | 202 (63.7) | 93 (13.9) | 75 (14.7) | 102 (9.2) | 58 (8.2) |
| Chronic obstructive pulmonary disease and bronchiectasis | 200 (16.7) | 157 (16.7) | 165 (32.9) | 99 (31.2) | 151 (22.6) | 91 (17.9) | 713 (64.2) | 437 (62.1) |
| Asthma | 237 (19.8) | 183 (19.4) | 140 (27.9) | 81 (25.6) | 97 (14.5) | 60 (11.8) | 596 (53.7) | 334 (47.4) |
| Aspiration pneumonitis; food/vomitus | 1 (0.1) | 4 (0.4) | 61 (12.2) | 32 (10.1) | 16 (2.4) | 17 (3.3) | 13 (1.2) | 3 (0.4) |
| Pleurisy; pneumothorax; pulmonary collapse | 64 (5.3) | 49 (5.2) | 60 (12.0) | 46 (14.5) | 86 (12.9) | 76 (14.9) | 98 (8.8) | 102 (14.5) |
| Respiratory failure; insufficiency; arrest | 125 (10.4) | 104 (11.1) | 211 (42.1) | 139 (43.8) | 276 (41.3) | 219 (43.0) | 607 (54.7) | 377 (53.6) |
| Cardiac and circulatory congenital anomalies | 137 (11.4) | 104 (11.1) | 160 (31.9) | 90 (28.4) | 293 (43.9) | 220 (43.2) | 76 (6.8) | 58 (8.2) |
| Digestive congenital anomalies | 47 (3.9) | 35 (3.7) | 51 (10.2) | 23 (7.3) | 110 (16.5) | 73 (14.3) | 92 (8.3) | 42 (6.0) |
| Genitourinary congenital anomalies | 120 (10.0) | 48 (5.1) | 120 (24.0) | 31 (9.8) | 153 (22.9) | 76 (14.9) | 128 (11.5) | 75 (10.7) |
| Nervous system congenital anomalies | 37 (3.1) | 21 (2.2) | 189 (37.7) | 129 (40.7) | 98 (14.7) | 64 (12.6) | 24 (2.2) | 18 (2.6) |
| Other congenital anomalies | 184 (15.3) | 140 (14.9) | 256 (51.1) | 168 (53.0) | 246 (36.8) | 179 (35.2) | 238 (21.4) | 151 (21.4) |
| Short gestation; low birth weight; and fetal growth retardation | 1 (0.1) | 4 (0.4) | 95 (19.0) | 47 (14.8) | 256 (38.3) | 202 (39.7) | 68 (6.1) | 40 (5.7) |
| Attention-deficit, conduct, and disruptive behavior disorders | 230 (19.2) | 122 (13.0) | 56 (11.2) | 21 (6.6) | 0 (0.0) | 0 (0.0) | 23 (2.1) | 8 (1.1) |
| Miscellaneous mental health disorders | 84 (7.0) | 89 (9.5) | 30 (6.0) | 24 (7.6) | 15 (2.2) | 11 (2.2) | 34 (3.1) | 26 (3.7) |
| Malignant cancer | 284 (23.7) | 232 (24.7) | 26 (5.2) | 14 (4.4) | 15 (2.2) | 21 (4.1) | 19 (1.7) | 10 (1.4) |
| Diabetis mellitus | 71 (5.9) | 86 (9.1) | 12 (2.4) | 4 (1.3) | 16 (2.4) | 24 (4.7) | 27 (2.4) | 21 (3.0) |
| Benign neoplasm | 95 (7.9) | 74 (7.9) | 37 (7.4) | 21 (6.6) | 41 (6.1) | 71 (13.9) | 69 (6.2) | 50 (7.1) |
| Essential hypertension | 149 (12.4) | 113 (12.0) | 35 (7.0) | 17 (5.4) | 27 (4.0) | 18 (3.5) | 10 (0.9) | 3 (0.4) |
| Infections | 824 (68.7) | 691 (73.4) | 377 (75.2) | 253 (79.8) | 492 (73.7) | 390 (76.6) | 1015 (91.4) | 660 (93.8) |

| | | | | | | | | |
|---|---|---|---|---|---|---|---|---|
| Other endocrine disorders | 434 (36.2) | 383 (40.7) | 231 (46.1) | 169 (53.3) | 198 (29.6) | 167 (32.8) | 277 (25.0) | 168 (23.9) |
| Hematologic disorders | 433 (36.1) | 329 (35.0) | 133 (26.5) | 88 (27.8) | 237 (35.5) | 166 (32.6) | 202 (18.2) | 116 (16.5) |
| Central nervous system infection | 23 (1.9) | 18 (1.9) | 45 (9.0) | 24 (7.6) | 30 (4.5) | 24 (4.7) | 23 (2.1) | 13 (1.8) |
| Eye disorders | 576 (48.0) | 462 (49.1) | 315 (62.9) | 198 (62.5) | 252 (37.7) | 210 (41.3) | 719 (64.8) | 446 (63.4) |
| Ear disorders | 569 (47.4) | 504 (53.6) | 271 (54.1) | 163 (51.4) | 199 (29.8) | 148 (29.1) | 833 (75.0) | 502 (71.3) |
| Heart diseases | 258 (21.5) | 190 (20.2) | 143 (28.5) | 92 (29.0) | 281 (42.1) | 205 (40.3) | 171 (15.4) | 105 (14.9) |
| Cerebrovascular disease | 58 (4.8) | 41 (4.4) | 121 (24.2) | 67 (21.1) | 38 (5.7) | 24 (4.7) | 17 (1.5) | 6 (0.9) |
| Circulatory disease | 174 (14.5) | 127 (13.5) | 115 (23.0) | 74 (23.3) | 113 (16.9) | 68 (13.4) | 39 (3.5) | 23 (3.3) |
| Upper respiratory disease | 974 (81.2) | 779 (82.8) | 442 (88.2) | 292 (92.1) | 567 (84.9) | 430 (84.5) | 1107 (99.7) | 703 (99.9) |
| Mouth disorders | 646 (53.8) | 503 (53.5) | 232 (46.3) | 134 (42.3) | 33 (4.9) | 34 (6.7) | 393 (35.4) | 296 (42.0) |
| Gastrointestinal disorders | 765 (63.8) | 606 (64.4) | 420 (83.8) | 259 (81.7) | 524 (78.4) | 375 (73.7) | 954 (85.9) | 597 (84.8) |
| Digestive system disease | 146 (12.2) | 112 (11.9) | 50 (10.0) | 25 (7.9) | 82 (12.3) | 51 (10.0) | 45 (4.1) | 28 (4.0) |
| Renal diseases | 115 (9.6) | 105 (11.2) | 41 (8.2) | 28 (8.8) | 92 (13.8) | 75 (14.7) | 21 (1.9) | 25 (3.6) |
| Urinary tract disorders | 320 (26.7) | 359 (38.2) | 157 (31.3) | 125 (39.4) | 192 (28.7) | 155 (30.5) | 185 (16.7) | 221 (31.4) |
| Genital disorders | 256 (21.3) | 166 (17.6) | 96 (19.2) | 32 (10.1) | 67 (10.0) | 13 (2.6) | 390 (35.1) | 128 (18.2) |
| Skin disorders | 486 (40.5) | 409 (43.5) | 213 (42.5) | 125 (39.4) | 187 (28.0) | 161 (31.6) | 560 (50.5) | 375 (53.3) |
| Artritis | 333 (27.8) | 322 (34.2) | 67 (13.4) | 50 (15.8) | 32 (4.8) | 20 (3.9) | 108 (9.7) | 55 (7.8) |
| Deformities | 470 (39.2) | 404 (42.9) | 174 (34.7) | 101 (31.9) | 29 (4.3) | 28 (5.5) | 170 (15.3) | 101 (14.3) |
| Perinatal trauma | 52 (4.3) | 43 (4.6) | 236 (47.1) | 148 (46.7) | 562 (84.1) | 380 (74.7) | 342 (30.8) | 158 (22.4) |
| Fractures and injuries | 802 (66.8) | 617 (65.6) | 302 (60.3) | 183 (57.7) | 198 (29.6) | 149 (29.3) | 758 (68.3) | 468 (66.5) |
| Mood and anxiety disorders | 243 (20.2) | 225 (23.9) | 22 (4.4) | 27 (8.5) | 71 (10.6) | 46 (9.0) | 31 (2.8) | 11 (1.6) |
| Developmental disorders | 307 (25.6) | 165 (17.5) | 365 (72.9) | 225 (71.0) | 54 (8.1) | 45 (8.8) | 136 (12.3) | 51 (7.2) |
